# Benefits of *Camelina sativa* Supplementation in Morphine Treatment: Enhanced Analgesia, Delayed Tolerance and Reduced Gut Side Effects Through PPAR-α Receptor Engagement

**DOI:** 10.3390/ijms26062519

**Published:** 2025-03-11

**Authors:** Elena Lucarini, Eleonora Pagnotta, Laura Micheli, Samuele Trisolini, Roberto Matteo, Laura Righetti, Alma Martelli, Lara Testai, Vincenzo Calderone, Lorenzo Di Cesare Mannelli, Carla Ghelardini

**Affiliations:** 1 Department of Neuroscience, Psychology, Drug Research, and Child Health—NEUROFARBA, Pharmacology and Toxicology Section, University of Florence, 50139 Florence, Italy; laura.micheli@unifi.it (L.M.); samuele.trisolini@unifi.it (S.T.); lorenzo.mannelli@unifi.it (L.D.C.M.); carla.ghelardini@unifi.it (C.G.); 2 CREA—Council for Agricultural Research and Economics, Research Centre for Cereal and Industrial Crops, 40128 Bologna, Italy; eleonora.pagnotta@crea.gov.it (E.P.); roberto.matteo@crea.gov.it (R.M.); laura.righetti@crea.gov.it (L.R.); 3 Department of Pharmacy, University of Pisa, 56126 Pisa, Italy; alma.martelli@unipi.it (A.M.); lara.testai@unipi.it (L.T.); vincenzo.calderone@unipi.it (V.C.); 4 Interdepartmental Research Centre Nutraceuticals and Food for Health—NUTRAFOOD, University of Pisa, 56126 Pisa, Italy; 5 Interdepartmental Research Centre of Ageing Biology and Pathology, University of Pisa, 56126 Pisa, Italy

**Keywords:** Brassicaceae, opioid tolerance, constipation, visceral hypersensitivity, glucosinolate, flavonoids, polyunsaturated fatty acids, peroxisome proliferator-activated receptor

## Abstract

Long-term opioid therapies are severely limited by the development of analgesic tolerance and gastrointestinal side effects. *Camelina sativa*, a plant of the Brassicaceae family, modulates the activity of peroxisome proliferator-activated receptor α (PPAR-α receptor), which is involved in the regulation of pain processing and gut physiology. The aim of this study was to evaluate the efficacy of *Camelina sativa* defatted seed meal (DSM) supplementation on the development of analgesic tolerance and side effects after repeated treatment with morphine in naïve mice. Co-administering *Camelina sativa* DSM (1 g kg^−1^ p.o.) and morphine (10 mg kg^−1^ s.c.) increased the efficacy and duration of the opioid-induced acute analgesic effect. Camelina supplementation also delayed the onset of tolerance to the morphine analgesic effect. The same result was obtained through either simultaneously administering morphine and camelina or administering camelina 24 h before morphine injection for the entire duration of the experiment. Camelina also counteracted intestinal damage and visceral hypersensitivity caused by morphine treatment. The beneficial effects of camelina on morphine-related analgesic efficacy and gut side effects were prevented via pre-treatment with the PPAR-α antagonist GW6471, though the latter did not influence the development of morphine tolerance. In conclusion, *Camelina sativa* DSM could be used as a supplement to improve the therapeutic profile of morphine.

## 1. Introduction

According to World Health Organization guidelines, opioids, like morphine, are the most effective treatment for pain, though their use is limited by their side effects, tolerance development and fears of addiction and dependence [1,2,3]. Analgesic tolerance is characterized by a reduced responsiveness to an opioid agonist which requires a progressive dose escalation to maintain the same efficacy, which also augments the risks of side effects, including nausea, vomiting, dizziness, sedation, itching, constipation and respiratory depression [4]. Constipation associated with abdominal pain is the side effect with the highest incidence, especially in the elderly. It is caused by the stimulation of µ opioid receptors located in the enteric neurons, which creates an overall inhibition of gastrointestinal secretion and motility, for which the body does not develop tolerance [5]. Osmotic laxatives and an increased intake of both fiber and liquids are recommended to prevent or treat constipation in patients before using peripherally acting µ-receptor antagonists. Indeed, though these antagonists can reduce constipation, their use is associated with relevant side effects in patients with concomitant gastrointestinal diseases [5,6]. Not least, chronic opioid use is associated with the establishment of physical dependence, attested by the occurrence of a withdrawal syndrome, which contributes to drug abuse and represents another major challenge to manage in patients [7]. Despite all these issues, the prevalence of opioids prescription for long-term use has risen in recent years, indicated in the number of patients presenting with an opioid use disorder [8,9]. The treatment of patients with chronic pain and opioid use disorder (estimated between 8% and 12% of patients with chronic non-oncological pain) is a complex clinical challenge because the tapering of opioids is likely to increase pain, whereas further increasing the opioid dose will aggravate the addiction, tolerance and pain sensitivity [10,11,12]. On the other hand, the clinical practice of opioid rotation, a strategy used to manage tolerance and side effects related to long-term opioids therapies, still needs to be optimized [13,14,15].

The limitations of the current available pharmacological treatments push the search for alternative approaches able to ameliorate the therapeutic and toxicological profile of opioids. Brassicaceae-derived glucosinolates and their hydrolysis-derived isothiocyanates were found to modulate the activity and the expression of several targets involved in pain regulation, including opioid-μ receptors, and to enhance the morphine analgesic effect [16]. *Camelina sativa* is a glucosinolate-containing plant from the Brassicaceae family that is particularly interesting due to its potential use in the production of functional foods [17,18]. Its seeds can be processed to obtain extracts or to produce flour enriched in active constituents such as glucosinolates, flavanols, flavonoids and polyunsaturated fatty acids, which have been found to exert protective effects on the nervous tissues and the bowel thanks to their antioxidant capacity [17] and the positive modulation of Peroxisome Proliferator-Activated Receptor-alpha (PPAR-α) [18]. It is worth noting that, on the one hand, oxidative stress contributes to the phenomenon of opioid tolerance, while on the other hand, PPAR-α is a molecular target of compounds able to counteract the development of opioid tolerance, such as palmitoylethanolamide [19]. Moreover, *Camelina sativa* has been shown to have a wide range of beneficial effects on the gut [17,18]. All this evidence makes *Camelina sativa* an excellent candidate to support opioid therapy.

Hence, the aim of the present work was to evaluate the efficacy of supplementation with *Camelina sativa* defatted seed meal (DSM) in enhancing analgesic effects, delaying the development of tolerance and counteracting the gastrointestinal toxicity associated with prolonged morphine treatment in mice. The mechanisms responsible for the beneficial effects of *Camelina sativa* DSM in this context have also been examined, with particular attention on the involvement of PPAR-α.

## 2. Results

### 2.1. Evaluation of the Effect of Camelina sativa DSM Acute Administration on Mice’ Pain Threshold and Morphine-Induced Antinociception

**Figure 1:**
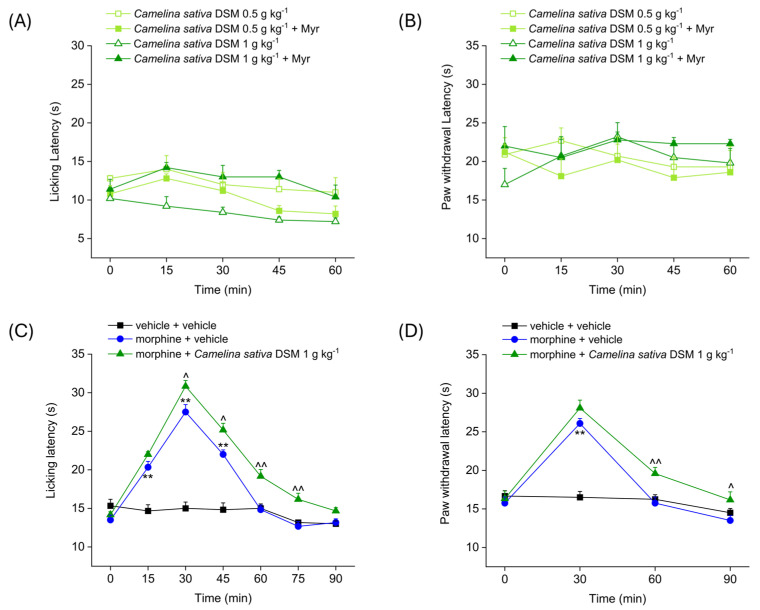
Effect of *Camelina sativa* DSM acute administration on mice pain threshold and morphine-induced antinociception. *Camelina sativa* DSM (0.5–1 g kg^−1^) with and without myrosinase (Myr; enzyme promoting bioactivation of glucosinolates) was suspended in 1X PBS and administered orally. The acute analgesic effect was measured by Hot Plate test (50 °C, (**A**)) and by Paw Pressure test (**B**). The data shown represent the mean ± SEM of 5 mice per experimental group. ** *p* < 0.01 vs. pre-test (0 min). Acute analgesic effect of co-administering morphine (10 mg kg^−1^ subcutaneously) and *Camelina sativa* DSM (1 g kg^−1^ per os, bioactivated with Myr). The analgesic effect was measured by Hot Plate test (49 °C, (**C**)) and by Paw Pressure test (**D**). The data shown represent the mean ± SEM of 6 mice per experimental group. Statistical significance was assessed by one- or two-way analysis of variance (ANOVA) with a Bonferroni post-test. ** *p* < 0.01 vs. vehicle + vehicle group. ^ *p* < 0.05 and ^^ *p* < 0.01 vs. morphine + vehicle group.

Figure 1 shows the analgesic effect of the acute administration of *Camelina sativa* DSM (0.5–1 g kg^−1^), before and after bioactivation with myrosinase (Myr), the enzyme that hydrolyzes glucosinolates to isothiocyanates. Antinociception was measured by Hot Plate and Paw Pressure tests. The Hot Plate test allows the evaluation of the mice’s response to a painful thermal stimulus, while the Paw Pressure test allows the evaluation of the mice’s response to a painful mechanical stimulus. Both tests were performed before (pre-test, 0 min) and at different times (15, 30, 45 and 60 min) after the acute administration of *Camelina sativa* DSM. The acute administration of increasing doses of *Camelina sativa* DSM did not elicit any analgesic effect in naïve mice, either before or after the bioactivation of the glucosinolates with myrosinase. Indeed, the mice’s pain threshold to both the thermal and mechanical pain stimulus (Figure 1A and Figure 1B, respectively) did not significantly change after the administration of *Camelina sativa* DSM. 

In the following experiment (Figure 1C,D), the effect of the co-administration of morphine and *Camelina sativa* DSM in naïve animals was evaluated. The Hot Plate test was performed before (pre-test, 0 min) and every 15 min (15, 30, 45, 60, 75 and 90 min) after the acute administration of *Camelina sativa* DSM (Figure 1C). The Paw Pressure test was performed before (pre-test) and every 30 min (30, 60 and 90 min) after the acute administration of *Camelina sativa* DSM (Figure 1D). The pain threshold of the mice treated with vehicle + vehicle remained unchanged throughout the duration of the test, whereas the pain threshold of mice treated with morphine was significantly increased compared to the pre-test. In both experimental groups treated with morphine, the analgesic effect occured 15 min after administration and peaked at 30 min. In mice treated with morphine + vehicle, the analgesic effect was exhausted at 60 min, whereas in the group of mice treated with morphine + *Camelina sativa* DSM, the analgesic effect was still significant 90 min after administration, both in the Hot Plate test (Figure 1C) and the Paw Pressure test (Figure 1D). Moreover, the analgesic effect of morphine + *Camelina sativa* DSM was significantly greater than that of the morphine + vehicle starting from 30 min (peak effect) up to 90 min after administration of the compounds.

### 2.2. Evaluation of the Effect of Camelina sativa DSM Supplementation on the Development of Tolerance to Analgesic Effect of Morphine

**Figure 2:**
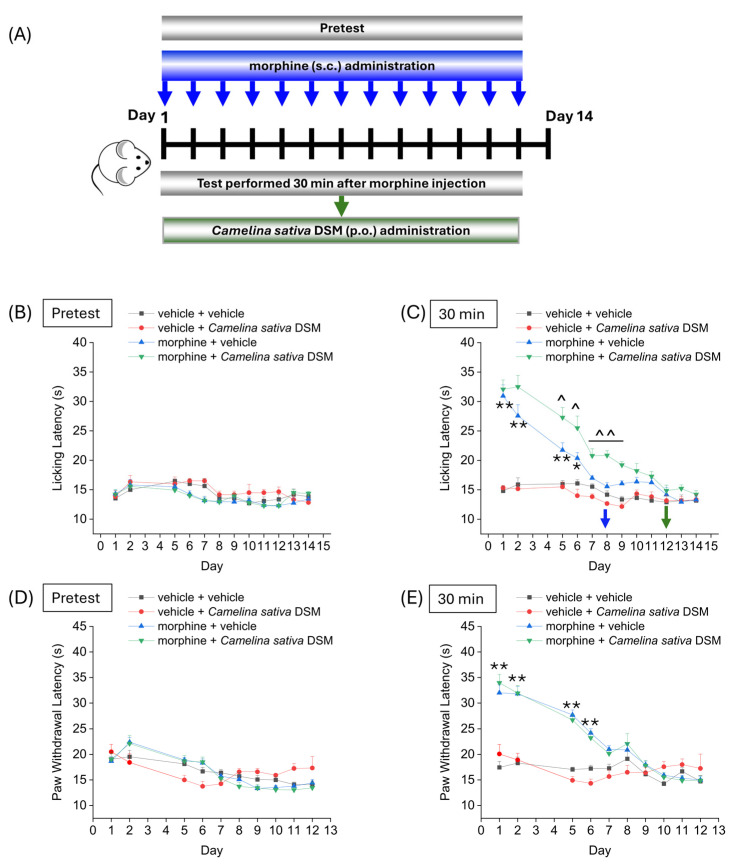
Effect of repeated administration of *Camelina sativa* DSM on the development of tolerance to the analgesic effect of morphine. Morphine (10 mg kg^−1^ subcutaneously) and *Camelina sativa* DSM (1 g kg^−1^ + Myr per os) were administered once daily in mice for 13 consecutive days according to the experimental scheme (**A**). Behavioral tests were performed daily 24 h after the administration of *Camelina sativa* DSM, before ((**B**,**D**); mice basal threshold) and 30 min after morphine injection in mice (**C**,**E**) by Hot Plate test (50 °C, (**B**,**C**)) and by Paw Pressure test (**D**,**E**). The data obtained represent the mean ± SEM per each experimental group (vehicle + vehicle, n = 11; vehicle + *Camelina sativa* DSM, n = 6; morphine + vehicle, n = 16; morphine + *Camelina sativa* DSM, n = 13–14). Statistical significance was assessed by one- or two-way analysis of variance (ANOVA) with a Bonferroni post-test. * *p* < 0.05 and ** *p* < 0.01 vs. vehicle + vehicle. ^ *p* < 0.05 and ^^ *p* < 0.01 vs. morphine + vehicle.

Figure 2 shows the effect of treatment with *Camelina sativa* DSM on the development of analgesic tolerance following repeated morphine administration. *Camelina sativa* DSM (1 g kg^−1^, bioactivated with Myr) and morphine (10 mg kg^−1^) were administered daily. Each day, the mice pain threshold was measured before (pre-test) and 30 min after the administration of morphine by Hot Plate test (B and C) and the Paw Pressure test (D and E), according to the scheme reported in Figure 2A. The repeated administration of vehicle + *Camelina sativa* DSM in naïve mice did not significantly influence mice basal pain threshold (Figure 2B,D) and showed no cumulative effect over time (Figure 2C,E). The group morphine + vehicle developed analgesic tolerance after 7 days of treatment (Figure 2B), while mice treated with morphine + *Camelina sativa* DSM developed analgesic tolerance after 11 days, as measured by the Hot Plate test (Figure 2B). Moreover, from day 5 to day 9, the analgesic effect of morphine administration was significantly greater in the mice treated with *Camelina sativa* DSM than in the mice receiving the vehicle (Figure 2C). In contrast, no significant differences were observed between the morphine + vehicle group and the morphine + *Camelina sativa* DSM group in the Paw Pressure test (Figure 2E).

### 2.3. Effect of Camelina sativa DSM and Morphine Co-Administration on the Development of Opioid-Related Analgesic Tolerance and Side Effects

**Figure 3:**
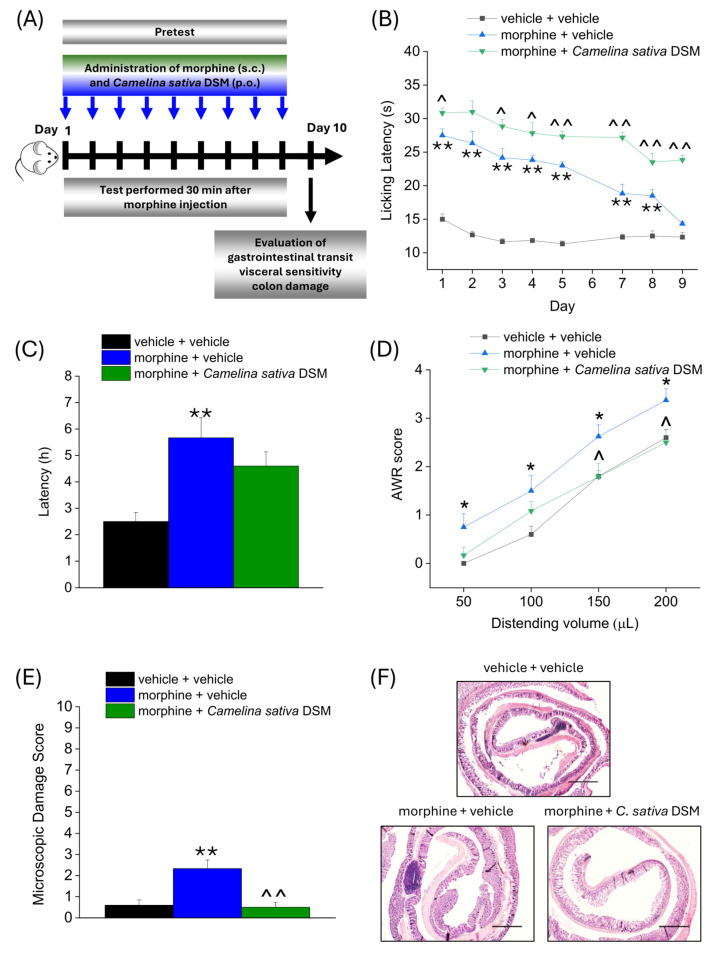
Effect of *Camelina sativa* DSM and morphine co-administration on the development of opioid-related analgesic tolerance and gastrointestinal side effects. Morphine (10 mg kg^−1^ subcutaneously) and *Camelina sativa* DSM (1 g kg^−1^ + Myr per os) were concomitantly administered in mice once daily for 9 consecutive days (experimental scheme, (**A**)). Behavioral tests were daily performed 30 min after the co-administration of *Camelina sativa* DSM and morphine in mice by Hot Plate test (50 °C, (**B**)). The gastrointestinal side effects were evaluated at the end of the behavioral tests, through the measure of the intestinal transit rate by the Carmine Red test, expressed as the average latency of excretion of the red-labeled pellet in the three experimental groups (**C**), the measure of mice visceral sensitivity by scoring (0–4) AWR response to colorectal distension (50–200 µL; (**D**)) and the analysis of microscopic damage to the colon (**E**), performed on H&E-stained slices (representative images in (**F**); Original magnification 4×; scale bar 500 μm). The data obtained represent the mean ± SEM of 6 mice per experimental group. Statistical significance was assessed by one- or two-way analysis of variance (ANOVA) with a Bonferroni post-test. * *p* < 0.05 and ** *p* < 0.01 vs. vehicle + vehicle group. ^ *p* < 0.05 and ^^ *p* < 0.01 vs. morphine + vehicle group.

According to the experimental scheme in Figure 3A, *Camelina sativa* DSM (1 g kg^−1^ + Myr) and morphine (10 mg kg^−1^) were co-administered starting from day 1 until the onset of morphine tolerance. Each day pain threshold was assessed 30 min after compounds administration by Hot Plate test. The acute analgesic efficacy of morphine + *Camelina sativa* DSM in naïve mice was significantly higher than that of morphine for the entire experiment (Figure 3B). On day 9, when tolerance developed in the group treated only with morphine (morphine + vehicle), the analgesic effect of morphine was still significant in the group co-administered with *Camelina sativa* DSM (Figure 3B).

On day 10, the three groups of mice were administered Carmine Red (6% in 1% CMC, 10 mL kg^−1^) in order to assess the intestinal transit rate. The average ejection latency of the first red pellet was then calculated (Figure 3C). The morphine + vehicle group showed a significantly higher latency than the control group (vehicle + vehicle). The morphine + *Camelina sativa* DSM group showed a mean red pellet expulsion latency that was approximately 1 h shorter than the morphine + vehicle group, although this effect did not reach statistical significance (Figure 3D).

The day after, visceral hypersensitivity (a symptom associated with the presence of altered gastrointestinal motility) was assessed in morphine-treated mice by measuring Abdominal Withdrawal Reflex (AWR score from 0 to 4) induced by colorectal distension (50–200 µL; Figure 3D). The morphine + vehicle group showed a significantly higher AWR score than the controls for all applied distention volumes. In the group treated with morphine + *Camelina sativa* DSM, the AWR score appeared lower than in the group treated with morphine + vehicle, though this difference reached statistical significance only with the highest distention volumes (150–200 µL; Figure 3D).

Colon samples were collected for histopathological analysis 24 h after completing behavioral assessments. Colon tissue sections were stained with hematoxylin and eosin (H&E) and scored using established criteria reported in the methods. In contrast to control (vehicle + vehicle) mice, morphine treatment led to a slight but significant increase in the microscopic damage score (Figure 3E), mainly driven by the influx of inflammatory cells into the intestinal tissue (Figure 3F; representative images), which was prevented by the supplementation with *Camelina sativa* DSM.

Other parameters were investigated during the treatment with *Camelina sativa* DSM and morphine (according to Supplementary Figure S1A). Food and water intake was not affected by the treatments (Supplementary Figure S1B,C). Although *Camelina sativa* DSM significantly reduced scratching behavior caused by morphine injection in mice, this effect was not statistically significant (Figure 1D). *Camelina sativa* DSM supplementation showed no effects on the morphine withdrawal crisis induced by the acute administration of naloxone (1 mg kg^−1^) and attested by the occurrence of specific behaviors in mice (chewing, head shakes, digging, exploring, wet dog shakes, rearing, cleaning, writing and loss of mobility; individual scores shown in Supplementary Figure S1). Indeed, the morphine + vehicle group showed a behavioral score significantly higher than that of the control group (vehicle + vehicle), but it was not different from that of the morphine + *Camelina sativa* DSM group (Supplementary Figure S1E).

### 2.4. Involvement of PPAR-α in Enhancing Antinociception and Reducing Gastrointestinal Side Effects of Morphine Related to Camelina sativa DSM Supplementation

**Figure 4:**
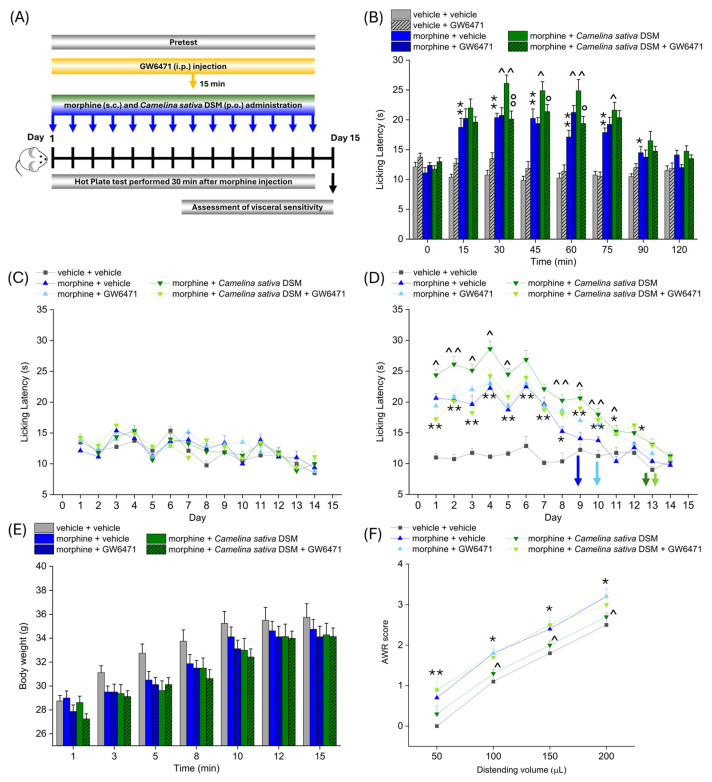
The involvement of PPAR-α in enhancing antinociception and reducing the gastrointestinal side effects of morphine related to *Camelina sativa* DSM supplementation. Morphine (10 mg kg^−1^ subcutaneously) and *Camelina sativa* DSM (1 g kg^−1^ + Myr per os) were concomitantly administered in mice once daily for 14 consecutive days. The selective antagonist of PPAR-α, GW6471 (2 mg kg^−1^, i.p.), was administered 15 min before *Camelina sativa* DSM and morphine, as described in the experimental scheme (**A**). The effect of PPAR-α antagonism on the increase in efficacy and duration of the morphine analgesic effect due to co-administration with *Camelina sativa* DSM was assessed on day 1 (**B**) by Hot Plate test (50 °C). The same behavioral test was repeated before ((**C**) pre-test) and 30 min after the daily co-administration of *Camelina sativa* DSM and morphine in mice (**D**) pre-treated or not with GW6471, until analgesic tolerance developed in all experimental groups. The tolerability of treatments was evaluated by monitoring body weight through all the experiments (**E**). At the end of the behavioral tests, mice visceral sensitivity was measured by scoring (0–4) the AWR response to colorectal distension (50–200 µL; (**F**)). The data obtained represent the mean ± SEM of 8 mice (or 6 mice for visceral pain assessment) per experimental group. Statistical significance was assessed by one- or two-way analysis of variance (ANOVA) with a Bonferroni post-test. * *p* < 0.05 and ** *p* < 0.01 vs. vehicle + vehicle group. ^ *p* < 0.05 and ^^ *p* < 0.01 vs. morphine + vehicle group. ° *p* < 0.05 and °° *p* < 0.01 vs. morphine + *Camelina sativa* DSM group.

According to the experimental scheme in Figure 3A, *Camelina sativa* DSM (1 g kg^−1^ + Myr) and morphine (10 mg kg^−1^) were co-administered starting from day 1 until the analgesic tolerance developed in all the experimetal groups. PPAR-α antagonist (GW6471; 2 mg kg^−1^) was injected in mice 15 min before morphine and *Camelina sativa* DSM. Each day, the pain threshold was assessed before (pre-test) and 30 min after morphine administration by Hot Plate test (experimental scheme in Figure 4A). The acute analgesic efficacy of the combined treatment morphine + *Camelina sativa* DSM in naïve mice was significantly higher than that of morphine (Figure 4B). This enhancement was not observed in the group receiving GW6471, which showed an effect similar to that of morphine + vehicle (Figure 4B). It is noteworthy that the administration of the PPAR-α antagonists did not influence morphine efficacy per se (Figure 4B).

No compound influenced the mice basal pain threshold after repeated treatments (Figure 4C). Morphine + *Camelina sativa* DSM was more effective than morphine + vehicle starting from day 1 up to day 9, when the group treated with morphine + vehicle developed analgesic tolerance. In the groups receiving *Camelina sativa* DSM, the onset of tolerance was postponed to day 13, irrespective of the administration of the PPAR-α antagonist GW6471 (Figure 4D). During the course of the experiment, a slight, but not significant, decrease in body weight was observed in all the groups receiving morphine (Figure 4E).

On day 15, the development of visceral hypersensitivity in morphine-treated mice (a symptom associated with the presence of altered gastrointestinal motility) was evaluated by measuring the Abdominal Withdrawal Reflex (AWR score from 0 to 4) induced by colorectal distension (50–200 µL; Figure 3D). The morphine + vehicle group showed a significantly higher AWR score than the controls for all applied distention volumes. The AWR score related to each distention volume was significantly lower in the group treated with morphine + *Camelina sativa* DSM with respect to that treated with morphine + vehicle (Figure 4F). The pre-treatment with the PPAR-α antagonist nullified the protective effect exerted by *Camelina sativa* DSM on the development of visceral hypersentitivity (Figure 4F).

Other parameters were investigated during the treatment with *Camelina sativa* DSM and morphine (according to Supplementary Figure S1A). Food and water intakes were not affected by the treatments (Supplementary Figure S1B,C). Although *Camelina sativa* DSM reduced the scratching behavior caused by morphine injection in mice, this effect was not statistically significant (Figure 1D). *Camelina sativa* DSM supplementation showed no effects on the morphine withdrawal crisis induced by the acute administration of naloxone (1 mg kg^−1^) and attested by the occurrence of specific behaviors in mice (chewing, head shakes, digging, exploring, wet dog shakes, rearing, cleaning, writing and loss of mobility; the individual scores are shown in Supplementary Figure S1). Indeed, the morphine + vehicle group showed a behavioral score significantly higher than that of the control group (vehicle + vehicle), but it was not different from that of the morphine + *Camelina sativa* DSM group (Supplementary Figure S1E).

## 3. Discussion

The present study highlighted the potential benefits provided by *Camelina sativa* DSM supplementation in long-term therapies with morphine. *Camelina sativa* can enhance the analgesic efficacy of morphine, delaying the onset of tolerance, and can counteract the development of gastrointestinal side effects. The positive modulation of PPAR-α emerged as the mechanism underlying its beneficial effects, apart from the tolerance delay.

As the number of non-terminally ill patients suffering from chronic pain increases [20,21], there is an urgent need for strategies to improve the therapeutic profile of opioids and to make their long-term employment in patients safe [4,9]. First, we observed that *Camelina sativa* DSM has no analgesic effects per se in naïve mice, neither before nor after bioactivation with the enzyme Myr, which is added to the suspension to convert the glucosinolates into isothiocyanates [22]. By contrast, the concomitant administration of morphine and *Camelina sativa* DSM can enhance the effect of the opioid and prolong its duration. At the therapeutic level, this implies that the co-administration of *Camelina sativa* could enable the reduction of the dose of morphine required to obtain a good therapeutic efficacy and, at the same time, reduce the number of administrations necessary to maintain the pain-relieving effect throughout the day, which might help in delaying the onset of both tolerance and side effects. Different actors and mechanisms might be responsible for these effects. We demonstrated that PPAR-α is mainly involved in the *Camelina sativa*-mediated enhancement of morphine efficacy. Camelina preparation contains different compounds able to influence the activity of this receptor, such as polyunsaturated fatty acids and flavonoids like naringenin, which can modulate the activation of PPAR-α [23,24,25,26], or isothiocyanates, which have been reported to act on the signaling pathways modulated by PPAR-α, such as those related to NF-kB [16,27,28]. Although the genetic ablation of PPAR-α has been reported to affect visceral nociception, likely because of biological adaptations, the blockade of PPAR-α does not affect mice sensitivity to pain [29]. The last finding is in line with our evidence showing no effect of the PPAR-α antagonist on the controls’ pain thresholds.

Regardless, we cannot exclude that other mechanisms contribute to determining a pharmacodynamic synergism with morphine. For instance, glucosinolates and isothiocyanates have been reported to act on different targets involved in pain, such as µ opioid receptors, increasing their expression and enhancing their analgesic effect [30,31,32]. Isothiocyanates are known for their capacity to behave as H_2_S donors and to activate Kv7 potassium channels [16,33], which might also contribute to opioid-induced pain relief [34]. Furthermore, *Camelina sativa* DSM has a high content of vitexin and naringenin, endowed with anti-inflammatory and neuroprotective properties [35,36]. Investigations into the anti-nociceptive properties of vitexin shed light on the multitarget nature of this flavonoid, which can modulate different receptors (opioid, GABA_A_, TRPV1) and processes (oxidative stress, cytokine production) involved in pain [35,37,38].

The pleiotropic effects of *Camelina sativa* gain further importance in long-term therapy. Indeed, we demonstrated that *Camelina sativa* DSM supplementation also counteract the development of tolerance to morphine, prolonging the duration of its analgesic effect by about 30% during chronic therapy, regardless of PPAR-α activation. Several tolerance mechanisms, such as neuroinflammation and glial activation [15,39,40], could be intercepted by the components of camelina extract [41,42,43], but further studies are needed to analyze the contribution of specific components and mechanisms. Potentially, camelina-derived flavonoids and isothiocyanates can regulate glial reactivity and inflammatory status [44,45,46]. Therefore, both the isothiocyanates and flavonoids contained in *Camelina sativa* DSM might contribute to the delay in the onset of morphine tolerance [18]. In this context, it is important to consider that the supplementation of *Camelina sativa* DSM elicited similar effects on morphine tolerance both when co-administered with the opioid and when its administration was deferred from that of morphine, a phenomenon which might involve the same or complementary mechanisms. *Camelina sativa* was instead not able to prevent or attenuate behavioral alterations related to morphine withdrawal crisis caused by naloxone injection in mice; this evidence indicates that camelina modulates opioid signaling through alternative mechanisms to those involved in the establishment of physical dependence.

In addition to tolerance and dependence, long-term therapies with opioids are responsible for the development of different side effects, including the slowing of intestinal transit. Unfortunately, this side effect is not tolerated, causing persistent abdominal pain and discomfort in patients, who develop a severe condition of constipation associated with intestinal irritation and tenderness [5]. Therefore, part of our study focused on evaluating the effect of supplementing camelina on the development of painful constipation in morphine-treated mice. The results obtained in this work demonstrated that *Camelina sativa* DSM is not able to significantly increase intestinal transit but can protect the colon from microscopic damage caused by the irritant condition of constipation, also attenuating the development of visceral hypersensitivity. Therefore, beyond ameliorating analgesic profile of opioids, the treatment with camelina can promote gastrointestinal health through the positive modulation of PPAR-α. This evidence adds to previous studies demonstrating the involvement of PPAR-α in the acute pain-relieving effect of *Camelina sativa* DSM in a model of colitis-associated visceral hyperalgesia. In the same model, camelina was also found to prevent colon damage and visceral pain after repeated treatments [18]. In this regard, it is interesting to also note that the methanolic and ethanolic extracts of *Camelina sativa* were reported to alleviate oxidative stress and symptoms in a mouse model of irritable bowel syndrome caused by stress exposure [17].

In addition to PPAR-α, other molecular targets could be involved in camelina-mediated beneficial gastrointestinal effects. In fact, the evidence in the literature demonstrated that preparations from other plants enriched in isothiocyanates, like *Eruca sativa*, can protect from the deleterious activation of enteric glia associated with intestinal inflammation, counteracting the establishment of visceral hypersensitivity [33,47]. Isothiocyanates are also endowed with prebiotic activity [48,49,50], which could contribute to maintaining intestinal well-being and counteracting the development of visceral hyperalgesia [51]. Moreover, the high content of flavonoids and fibers in *Camelina sativa* could bring benefits for intestinal health [52], involving the microbiota [53,54]. Interestingly, the gut microbiome has been found to play a critical role in opioid tolerance, with opioids causing the dysbiosis of the gut, and changes in the gut microbiome impacting opioid tolerance [55].

A recent work correlated suppressed muscle contractility, increased neuronal excitability and visceral hypersensitivity in morphine-treated rats to the upregulation of COX-2 and nerve growth factor (NGF) in the colon smooth muscle [56]. Given the contribution of NGF to neurotoxicity and pain [57], the prevention of morphine-related visceral hypersensitivity could be attributable to a protective effect of *Camelina sativa* DSM on enteric neurons [18], though further studies are needed to clarify this aspect. Considering all these potential mechanisms of action, the beneficial effects of *Camelina sativa* DSM on the colon are probably due to a pharmacological synergism between its different components.

In conclusion, the results of this study suggest that supplementation with *Camelina sativa* DSM might improve the therapeutic profile of opioids, though the same efficacy needs to be proven in a disease context. It is worth noting that *Camelina sativa* DSM can be regarded as nutraceutical and thus used for the development of either food supplements or functional foods, increasing patient compliance and tolerability. In this regard, it is important to reiterate that in the present study, as in our previous published work [18], no side effects have been observed in animals (mice or rats) after repeated treatment with *Camelina sativa* (an edible plant), attesting to the safety of the compound and the chosen dose.

## 4. Materials and Methods

### 4.1. Mice

Two-month-old male CD-1 mice (Envigo, Varese, Italy) weighing 20–25 g at the beginning of the experimental procedure were used. The mice were housed in the Centro Stabulazione Animali da Laboratorio (University of Florence) and used at least 1 week after their arrival. Ten mice were housed per cage (size 26 cm × 41 cm); the mice were fed a standard laboratory diet and tap water ad libitum and kept at 23 ± 1 °C with a 12 h light/dark cycle (light at 7 a.m.). Food and water intake was monitored throughout the experiment.

All animal manipulations were carried out according to the Directive 2010/63/EU of the European Parliament and of the European Union Council (22 September 2010) on the protection of animals used for scientific purposes. The ethical policy of the University of Florence complies with the Guide for the Care and Use of Laboratory Animals of the US National Institutes of Health (NIH Publication No. 85-23, revised 1996; University of Florence assurance number: A5278-01). Formal approval to conduct the experiments described was obtained from the Italian Ministry of Health (No. 498/2017) and from the Animal Subjects Review Board of the University of Florence. Experiments involving animals have been reported according to ARRIVE guidelines [58]. All efforts were made to minimize animal suffering and to reduce the number of animals used.

### 4.2. Treatments

*Camelina sativa* defatted seed meal (DSM) was produced at the Council for Agricultural Research and Economics—Research Centre for Cereal and Industrial Crops (CREA-CI; Bologna, Italy) and characterized by moisture (4.4%), protein content (35% *w*/*w* on dry matter), residual oil content (21.5% *w*/*w* on dry matter, with a Ω 6/Ω 3 ratio of 0.67 and characterized by 33% alpha linolenic acid, 19% linoleic acid and 17% oleic acid as the main components accounting for about 70% of total fatty acids) and total glucosinolates (33 ± 2 µmol g^−1^) according to previously published procedures [18,59]. The potential analgesic effect of *Camelina sativa* DSM (0.5, 1 g kg^−1^) was evaluated before and after bioactivation with myrosinase (23.4 U mL^−1^), and isolated from ripe seeds of *Sinapis alba* L. according to Pessina et al. [60]. *Camelina sativa* DSM was suspended in 1X PBS and administered into naïve mice orally (15 mL kg^−1^). For the bioactivation of glucosinolates, the enzyme myrosinase (Myr) was added to the suspension and incubated for 30 min (at 37 °C under gentle agitation) before administration [18]. Morphine (10 mg kg^−1^; Molteni Farmaceutici, Scandicci, Italy) was dissolved in physiological saline and administered subcutaneously (10 mL kg^−1^). The potential synergism of *Camelina sativa* DSM on the analgesic effect of morphine was evaluated in the same mice acutely treated with both compounds. Subsequently, the effect of *Camelina sativa* DSM on the development of analgesic tolerance to morphine was evaluated. *Camelina sativa* was orally administered at a dose of 1 g kg^−1^, after bioactivation with myrosinase, while morphine 10 mg kg^−1^ was administered subcutaneously, once daily. The control group (vehicle + vehicle) received the vehicles of morphine and *Camelina sativa* DSM. The selective PPAR-α antagonist GW6471 (2 mg kg^−1^, i.p.; MedChemExpress, Monmouth Junction, NJ, USA) was dissolved in saline + 10% DMSO + 5% Tween20 and administered daily in mice 15 min before the administration of morphine and *Camelina sativa* DSM. The analgesic effect was assessed 30 min after morphine administration, both when *Camelina sativa* and morphine were administered together and deferred. The treatment was repeated until analgesic tolerance developed in all the experimental groups.

### 4.3. Hot Plate Test

Antinociception was assessed using the Hot Plate test. With minimal animal–handler interaction, mice were taken from home cages and placed onto the surface of the hot plate (Ugo Basile, Varese, Italy) maintained at a constant temperature of 50 °C  ±  1 °C. Ambulation was restricted by a cylindrical Plexiglas chamber (diameter, 10 cm; height, 15 cm), with an open top. A timer controlled by a foot peddle began timing response latency from the moment the mouse was placed onto the hot plate. Pain-related behavior (licking of the hind paw) was observed and the time (seconds) of the first sign was recorded. The cutoff time of the latency of paw lifting or licking was set at 40 s [61].

### 4.4. Paw Pressure Test

Paw withdrawal latency (in seconds) to mechanical stimulation (applied to paw dorsal surface) was assessed with the Paw Pressure test and used as a measure of mechanical pain threshold [62]. A 15 g calibrated glass cylindrical rod (diameter = 10 mm) chamfered to a conical point (diameter = 3 mm) was used to exert the mechanical force. The weight was suspended vertically between 2 rings attached to a stand and was able to freely move vertically. A single measure was made per animal. A cutoff time of 40 s was used.

### 4.5. Assessment of Intestinal Transit Time

To study intestinal transit, Carmine Red assay was used as described by Koester et al. [63]. Briefly, 250 µL of a sterilized 6% (*w*/*v*) solution of Carmine Red dye (Sigma-Aldrich, Milan, Italy) in 1% CMC (Sigma-Aldrich) was delivered per mouse via oral gavage. Fecal output was monitored every 30 min or more frequently if the stool passed spontaneously. The time from gavage to the appearance of the bright red dye was recorded as the whole intestinal transit time.

### 4.6. Assessment of Visceral Sensitivity by Abdominal Withdrawal Reflex

The behavioral responses to Colon–Rectal Distension (CRD) were assessed via Abdominal Withdrawal Reflex (AWR) measurement using a semiquantitative score in conscious mice [64]. Briefly, mice were anesthetized with isoflurane, and a lubricated latex balloon, attached to polyethylene tubing, assembled into an embolectomy catheter and connected to a syringe filled with water, was inserted through the anus into the rectum and descending colon. The tubing was taped to the tail to hold the balloon in place. The mice were then allowed to recover from anesthesia for 30 min. The AWR measurement consisted of the visual observation of mouse responses to graded CRD (50, 100, 150, 200 µL) by blinded observers who assigned an AWR score: no behavioral response to CRD (0); immobility during CRD and occasional head clinching at stimulus onset (1); mild contraction of the abdominal muscles but no abdominal lifting from the platform (2); strong contraction of the abdominal muscles and lifting of the abdomen off the platform (3); arching of the body and lifting of the pelvic structures and scrotum (4).

### 4.7. Histological Analysis of Colon

The presence of colon damage was investigated ex vivo in accordance with the methods used in previous studies [65]. For the histological analysis, the colon was fixed in 4% paraformaldehyde for 24 h, dehydrated in alcohol, included in paraffin and cut into 5 μm sections. The microscopic evaluations of colon damage (mucosal architecture loss, cellular infiltrate, muscle thickening, crypt abscess and goblet cell depletion) were carried out on hematoxylin/eosin-stained sections by two blind investigators. Representative digitalized images were collected by a Leica DMRB light microscope equipped with a DFC480 digital camera (40× magnification; Leica Microsystems, Wetzlar, Germany).

### 4.8. Withdrawal Paradigms

Withdrawal was assessed 12 h after the last injection; that is, the morning after the previous day’s afternoon dose. To evoke precipitated withdrawal, mice were intraperitoneally injected with naloxone 2 mg/kg, placed in individual cages and consequently observed for 30 min. Withdrawal signs were interpreted through analyzing several parameters and using a modified score from Uddin et al. [66], with minor modifications. The analyzed signs were the following: chewing (2), head shakes (2), digging (1), exploring (1), wet dog shakes (2), rearing (2), cleaning (2), writing (2) and loss of mobility (2). The total score for each mouse ranged between 0 (normal mice) and 16 points (withdrawal sign) [67].

### 4.9. Acute Itch

On the day of behavioral testing, mice were individually placed in small plastic chambers (15 × 15 × 15 cm^3^) on an elevated metal mesh floor and allowed at least 30 min for habituation. Mice were given an s.c. injection of morphine (10 mg kg^−1^) and the oral administration of *Camelina sativa* DSM (1 g kg^−1^). After the injection, the time spent scratching was quantified for 30 min. A scratch behavior was considered when the mouse lifted its hind paw to scratch and returned the paw to the floor [68].

### 4.10. Statistical Analysis

All measurements were made by researchers blinded to the mouse treatments. Data were analyzed using “Origin 9” software (OriginLab, Northampton, MA, USA) by one- or two-way analysis of variance (ANOVA) with a Bonferroni post-test, with *p* < 0.05 or 0.01 considered statistically significant, respectively. The results were shown as means ± the standard error of the mean (SEM) of n assessments, depending on the experiment.

## Data Availability

Data is contained within the article and Supplementary Materials.

## Supplementary Materials

The following supporting information can be downloaded at https://www.mdpi.com/article/10.3390/ijms26062519/s1.
