# Supplementary material for: Benefits of Camelina sativa Supplementation in Morphine Treatment: Enhanced Analgesia, Delayed Tolerance and Reduced Gut Side Effects Through PPAR-α Receptor Engagement
Source: Int J Mol Sci. 2025 Mar 11;26(6):2519. doi: 10.3390/ijms26062519 (PMC11942378; doi:10.3390/ijms26062519)
Supplement: Supplementary file 1 [file ijms-26-02519-s001.zip › ijms-3458426-supplementary.pdf]

Supplementary Materials

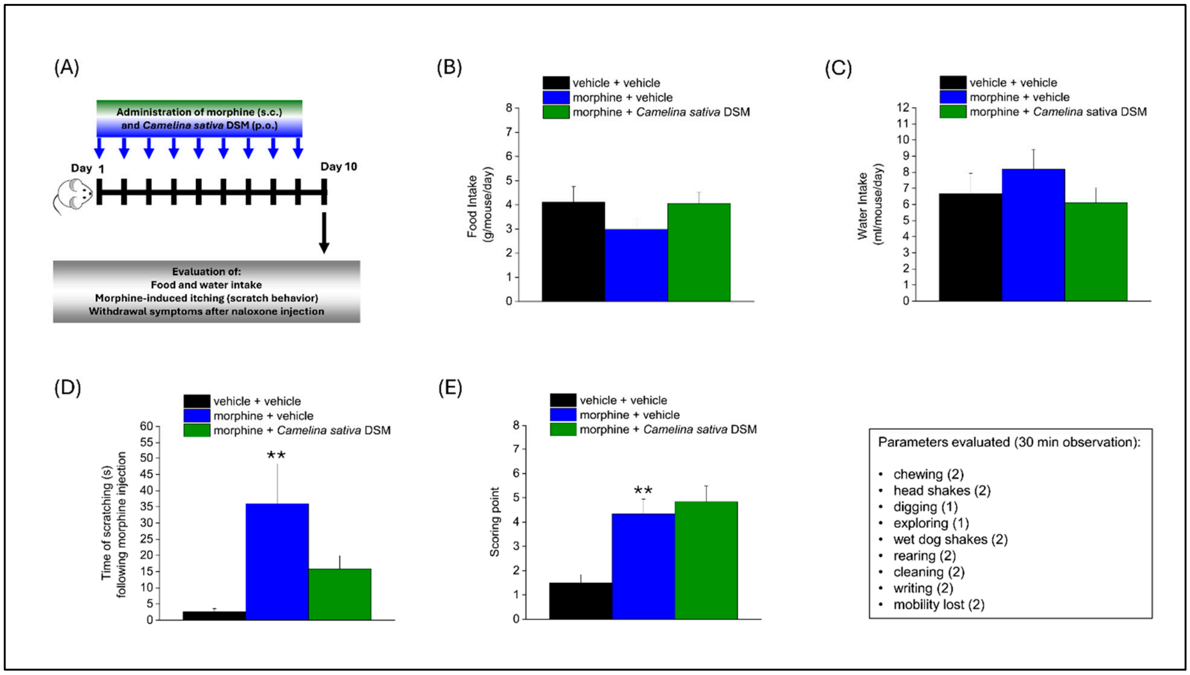

Figure S1. Evaluation of *Camelina sativa* DSM effect on altered food/water intake, itching and withdrawal symptoms induced by acute injection of naloxone in morphine-treated animals. Morphine (10 mg kg^{-1} subcutaneously) and *Camelina sativa* DSM (1 g kg^{-1} + Myr *per os*) were administered once daily in animals for 9 consecutive days (**A**). Food and water intake have been monitored in the animals for the entire experiment (**B**,**C**). On day 9, the scratching time after morphine injection was used as a measure of itching (**D**). On day 10, the presence of withdrawal-related symptoms after acute administration of naloxone (1 mg kg^{-1}) was assessed in each experimental group. A score was associated with each symptom: chewing (2), head shakes (2), digging (1), exploring (1), wet dog shakes (2), writhing (2), rearing (2), cleaning (2), mobility lost (2). The total score of each animal was used as a direct measure of the extent of naloxone-induced opioid withdrawal (E). The data obtained represent the mean ± SEM of 6 animals per experimental group. ** $p < 0.01$ vs vehicle + vehicle group.
